# Large airway-obstructing retropharyngeal lipoma in an asymptomatic patient: a case report

**DOI:** 10.1186/s40729-020-00237-3

**Published:** 2020-08-15

**Authors:** Scott A. Ehlers, John M. Bozanich, Mehrnaz Tahmasbi Arashlow, Hui Liang, Madhu K. Nair

**Affiliations:** 1 Division of Oral and Maxillofacial Radiology, Department of Diagnostic Sciences, Texas A&M College of Dentistry, 3302 Gaston Avenue, Dallas, TX 75246-0677 USA; 2 Department of Periodontics, Texas A&M College of Dentistry, Dallas, TX USA

**Keywords:** Lipoma, CBCT, MDCT, MRI, OMFR, Retropharyngeal

## Abstract

**Background:**

Lipomas are common benign mesenchymal tumors that appear in the head and neck region in approximately 25% of cases where they are noted. Lipomas of the airway region are exceedingly rare, accounting for less than 1% of airway obstruction tumors. Correlation of radiographic findings from cone beam computed tomography (CBCT), multi-detector computed tomography (MDCT), and magnetic resonance imaging (MRI) of a rare retropharyngeal lipoma has not been previously reported. CBCT studies acquired for implant and/or other diagnostic purposes may be the first line of detection as an incidental finding.

**Case presentation:**

A 66-year-old female presented for a pre-implant CBCT with no history of other complaints or signs/symptoms. CBCT imaging depicts a large, well-defined, low-attenuation/soft tissue density lesion with an undulating appearance extending from the posterior left pharyngeal wall and occluding two-thirds of the airway from C2 to C4. The lesion extends laterally into the left parapharyngeal space and inferiorly beyond the field of view of the study. Evidence of faint internal septations was noted. The patient was immediately referred for an ENT consult. Laryngoscopy, MRI, and contrast-enhanced MDCT imaging were conducted to determine the full extent and nature of the lesion, as well as to potentially plan for biopsy and/or surgical resection. Removal of the lesion was successful, and histopathologic evaluation confirmed lipoma. Periodic follow-up was recommended to monitor for possible recurrence.

**Discussion:**

The slower growth pattern of some benign lesions may obscure any symptoms as changes the patient may normally notice take place over an extended period. Furthermore, soft tissue lesions and especially those in the posterior midline, such as in this case, may not be easily visible on routine panoramic imaging or clinical exam, allowing for substantially large growth before detection. While the soft tissue contrast of the CBCT volume is poor, enough information was present to establish an initial differential diagnosis and the need for more advanced imaging modalities. With the growing popularity and adoption of CBCT in maxillofacial imaging, a thorough understanding of the appearance of hard and soft tissue lesions, as well as a strong understanding of the baseline appearance of normal anatomy, is important to ensure no incidental pathoses go undiagnosed.

## Background

Lipomas are relatively common benign mesenchymal tumors that are usually found in the torso and extremities. Lipomas in the head and neck region are more uncommon, accounting for 25% of all lipomas [1]. Lipomas of the retropharyngeal spaces are quite rare, accounting for less than 1% of all benign neoplasms in the airway [2]. Of the few cases reported in a search of the literature, symptoms such as obstructive sleep apnea or swelling were reported, leading to the detection of the tumor [3–7]. In the case presented in this report, no symptoms of any kind were reported.

Additionally, the cases reported previously demonstrate the appearance of retropharyngeal lipomas in contrast-enhanced MDCT (multi-detector computed tomography) and MRI (magnetic resonance imaging) [3–7]. However, the initial discovery of this tumor was via a routine pre-surgical CBCT (cone beam computed tomography). The description of the appearance of a retropharyngeal lipoma on CBCT, which lacks soft tissue definition and contrast, has not been previously reported. Juxtaposition of CBCT, MDCT, and MRI is useful for correlation with its appearance on CBCT. It also demonstrates the need for advanced imaging such as MDCT and MRI to further characterize and outline the lesion for appropriate management.

This case report aims to demonstrate the characteristics of a retropharyngeal lipoma on CBCT, MDCT, and MRI and to demonstrate the importance of reading the CBCT examination in full so that appropriate intervention can be instituted. Furthermore, it highlights the rather insidious growth potential of tumors in the retropharyngeal space without pronounced symptoms. Additionally, this report serves to emphasize that a thorough understanding of the radiographic appearance of anatomic structures as seen on CBCT (or any other imaging modality) is imperative to identifying critical incidental findings with potential serious consequences if left untreated such as an airway obstruction.

## Case presentation

**Fig. 1:**
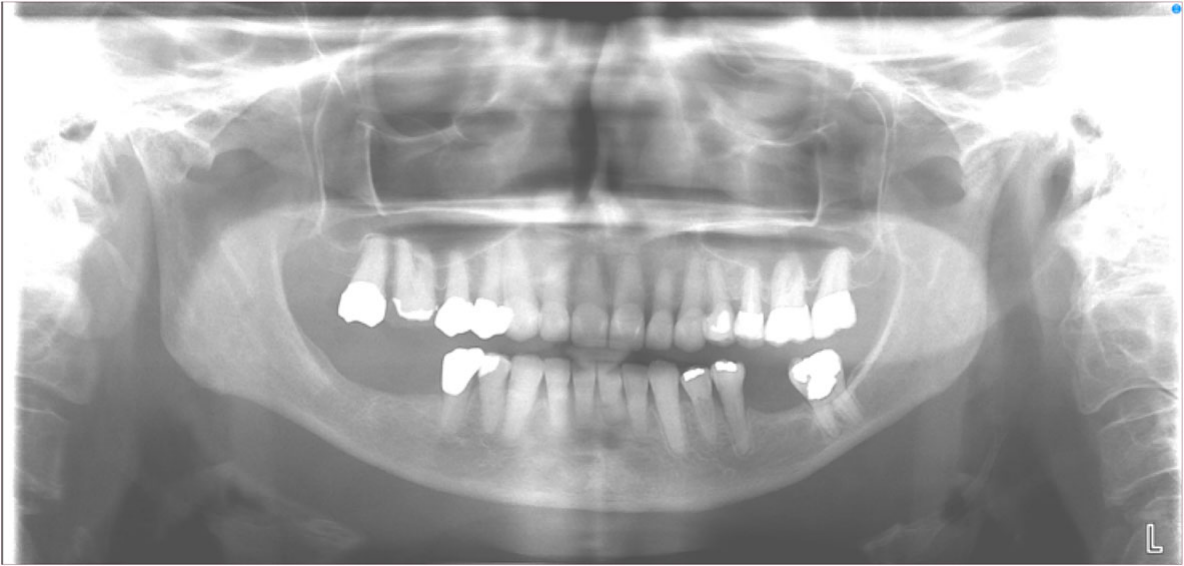
Panoramic radiograph taken 15 months prior to initial CBCT study. The brightness has been increased to highlight soft tissue anatomy. The lesion is not immediately apparent on the radiograph

A 66-year-old female was seen for potential implant treatment. Clinical examination revealed missing posterior mandibular teeth intra-orally with no significant extra-oral findings. The patient’s chief complaint was her partial edentulism which she wanted to address via implants. The previous panoramic radiograph taken 15 months prior was reviewed with no significant abnormalities noted (Fig. 1). The patient received an initial, preoperative CBCT examination for presurgical treatment planning including localization of critical anatomic structures such as the inferior alveolar canal.

**Fig. 2:**
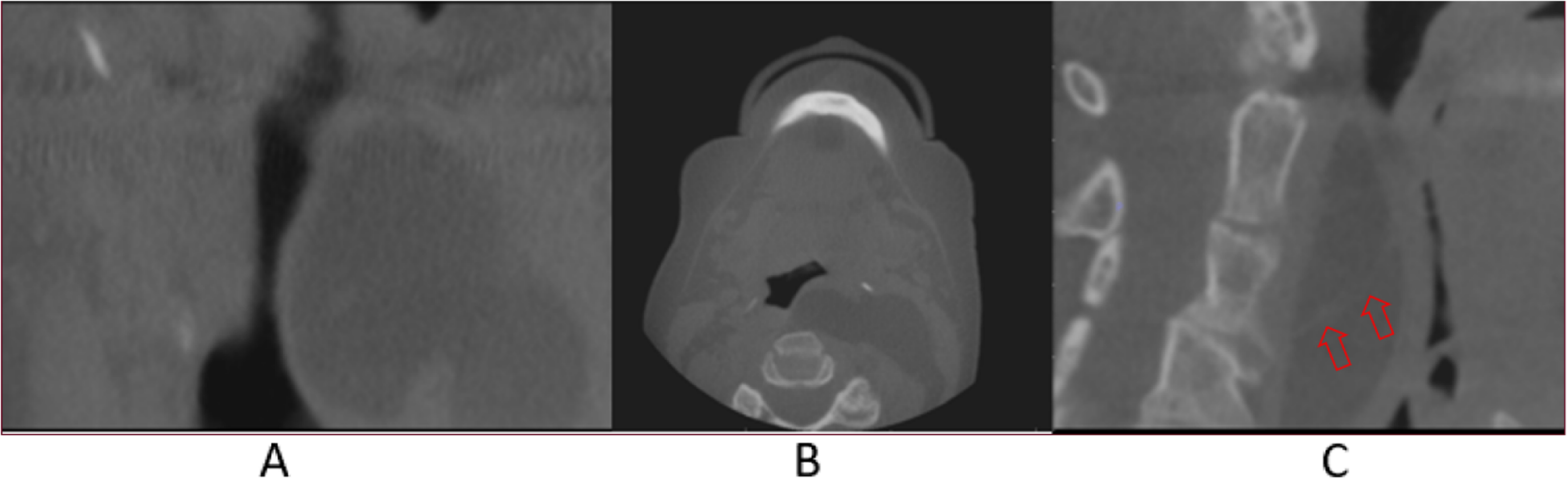
CBCT coronal (**a**), axial (**b**), and sagittal (**c**) images showing the soft tissue density lesion with faint internal septation (arrows). The full extent of the lesion was not initially within the field of view. No obvious change to the external soft tissue contours can be seen

The CBCT study was acquired (iCAT FLX, Imaging Sciences International LLC, Hatfield, PA 19440, USA) extending from the level of the dens to that of C4 with the following scan parameters: 120 kVp, 5 mA, voxel size 0.3 mm, scan time 8.9 s, and field of view 16 × 6 cm. The CBCT volume (Fig. 2) depicted a large, well-defined, low-attenuation/soft tissue density lesion with an undulating and lobulated appearance extending from the posterior left pharyngeal wall and occluding two-thirds of the airway from C2 to C4. The lesion extended laterally into the left parapharyngeal space and extended inferiorly beyond the field of view of the initial CBCT study. The lesion appeared to approach the level of the skull base superiorly in the left parapharyngeal space. Evidence of faint internal septations was noted. No evidence of asymmetry of the external soft tissue contours of the head and neck region was noted.

The lack of symptoms along with the significant displacement but absence of perforation to involved surrounding structures suggested a benign but fairly aggressive growth pattern. The initial differential radiographic diagnosis included a lipoma as well as more common benign tumors found in the retropharyngeal area such as minor salivary gland, vascular, and neural tumors. The patient was immediately referred for an ENT consult.

**Fig. 3:**
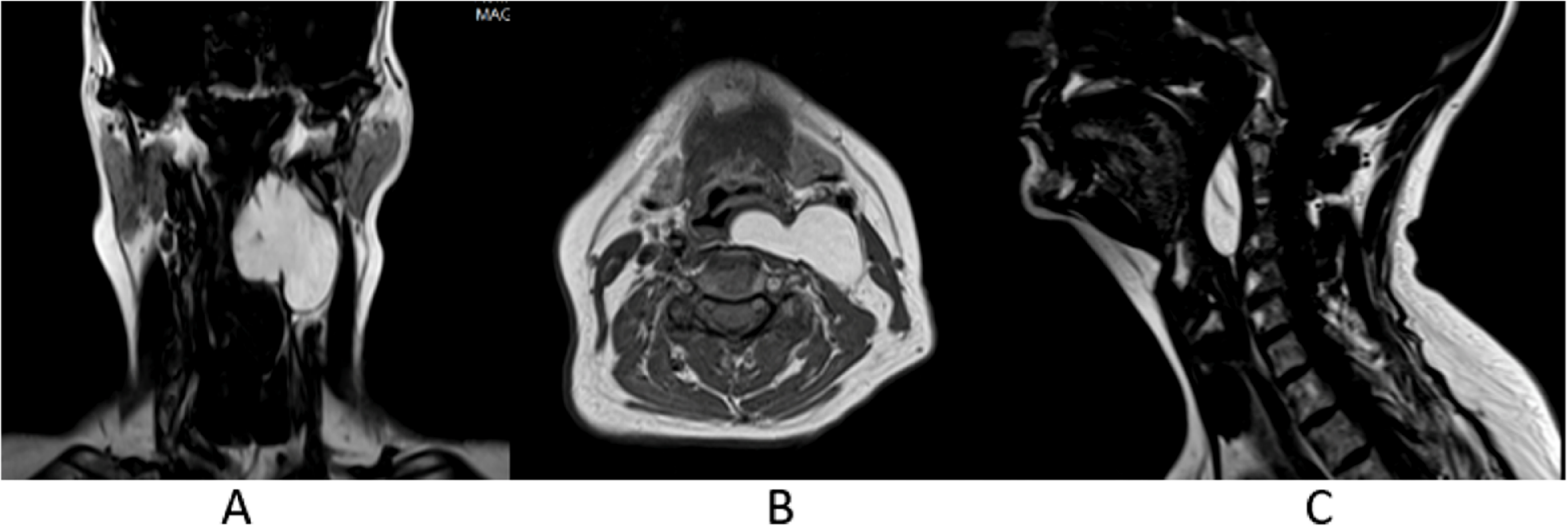
MRI T1-weighted coronal (**a**), axial (**b**), and sagittal (**c**) images showing strong enhancement of the lesion. Impingement on the airway as well as evidence of internal septation can be readily seen

**Fig. 4:**
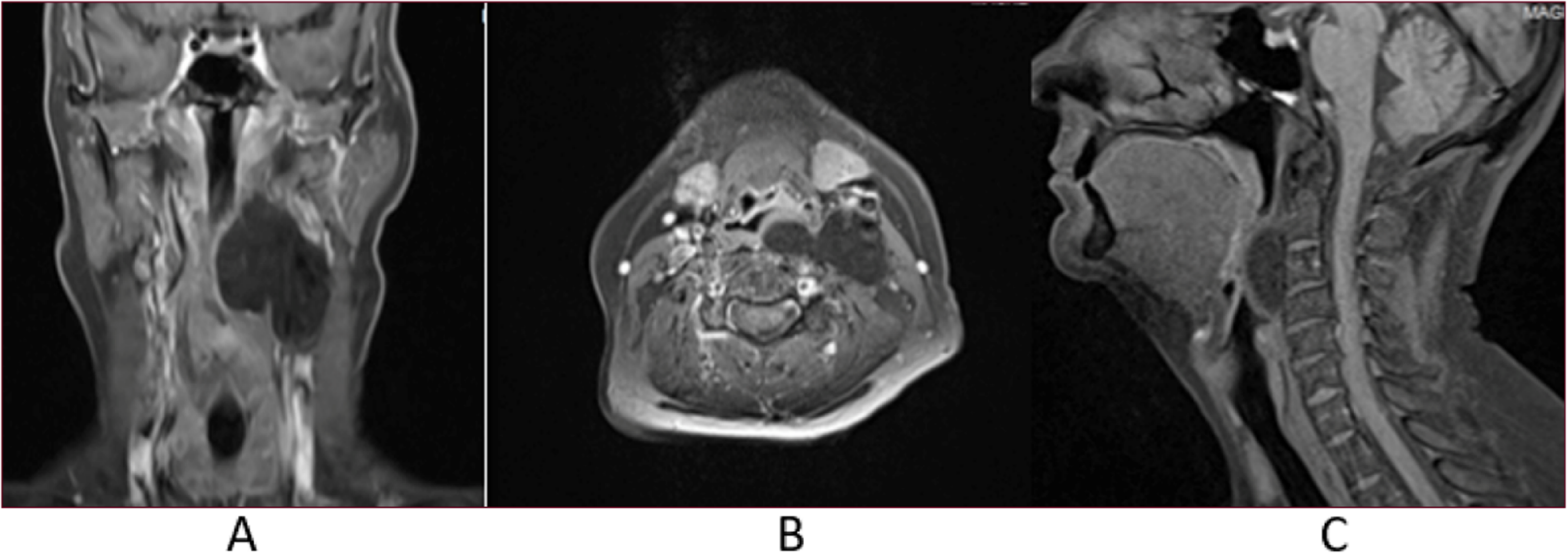
MRI T1-weighted with fat suppression coronal (**a**), axial (**b**), and sagittal (**c**) images depicting a homogeneous loss of signal within the lesion

**Fig. 5:**
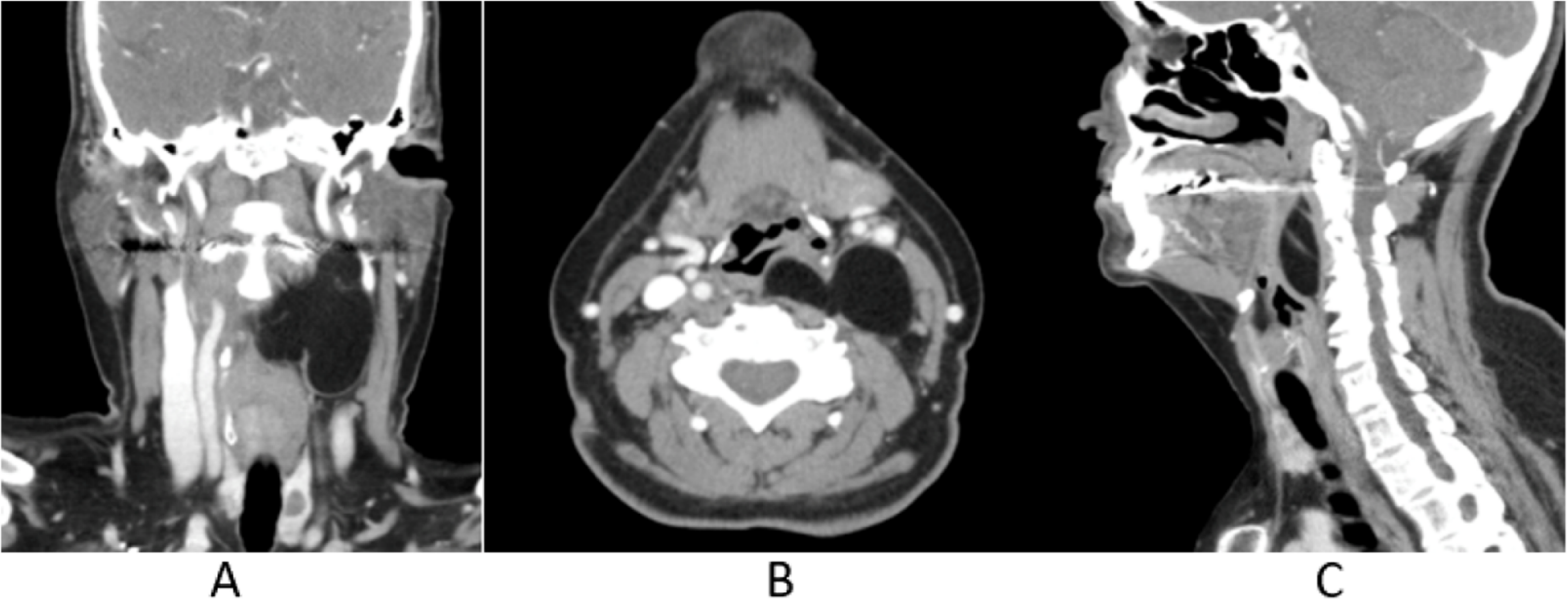
Soft tissue window contrast-enhanced MDCT coronal (**a**), axial (**b**), and sagittal (**c**) images depicting a large, lobulated mass in the soft tissue of the neck impinging on the airway. Septation within the lesion can be seen in the sagittal view

Following the ENT consult, laryngoscopy, MRI, and contrast-enhanced MDCT imaging were conducted to determine the full extent of the lesion and to plan for potential surgical intervention. MRI (Figs. 3 and 4) and contrast-enhanced MDCT (Fig. 5) demonstrated the inferior extent of the lesion as well as provided superior soft tissue contrast and imaging features that allowed for a narrowing of the differential diagnosis. Specifically, the homogeneously hyperintense signal noted throughout the lesion using T1-weighted MRI protocols (Fig. 3) in conjunction with the homogeneously hypointense signal noted when using T1 with fat suppression protocols (Fig. 4) confirmed the initial top differential diagnosis choice of benign lipoma.

On both MRI (Figs. 3 and 4) and contrast-enhanced MDCT (Fig. 5), displacement of the surrounding structures within the soft tissue was apparent including the significant anterior displacement of the carotid sheath. Additionally, with a larger field of view, further confirmation that there was no obvious displacement of the external contours of the head and neck was possible.

Treatment was conducted under general anesthesia. A 5-cm transverse incision was made in the left upper neck. The mass was found behind the carotid sheath and approached by going posterior to the carotid sheath to dissect from the retropharyngeal tissue and the longus colli. The lesion extended superiorly to the skull base. The entire mass was completely removed without rupture. A 20-g specimen from the resection was sent for biopsy. The specimen was described as yellow-tan to slightly yellow-brown and found to be mature adipose tissue, consistent with lipoma. After sufficient recovery time, the patient was able to proceed with the initial treatment plan including bone grafting and is pending final implant placement.

## Discussion

Understanding the common and uncommon locations where a lipoma may present is important whether conducting a clinical or radiographic examination of a patient. As previously stated, presentation of lipomas in the head and neck region accounts for approximately 25% of cases. Of those cases, the majority are located in the superficial tissue of the neck, typically in the posterior triangle [1]. Lipomas located in the deep tissue of the head and neck are much more uncommon with most deep lipomas found in the forehead or scalp region [8]. Recognizing the appearance of a benign lipoma in CBCT imaging is important as CBCT imaging becomes more widely adopted in the dental and other medical fields. While soft tissue contrast in CBCT scan volumes is typically poor, differentiation of the internal structure of the tumor as lower attenuating as compared to the soft tissue of the retropharyngeal wall and higher attenuating than the airway itself was possible. Additionally, the attenuation of the septations within the lesion was similar to that of the soft tissue of the posterior pharyngeal wall. The attenuation of the main body of the tumor was similar to the various fat planes best seen in the axial view.

As demonstrated in this case, tumors of the retropharyngeal space and other deep tissue areas may grow to considerable size with no appreciable symptoms. Additionally, lesions located in the posterior mid sagittal plane may not be readily apparent on routine panoramic radiographs, allowing for lesions in this area to grow to considerable size before detection. In this case, the panoramic radiograph taken approximately 15 months prior to discovery of the lipoma demonstrated no signs of the lesion (Fig. 1).

With the increasing use of CBCT for dental implant planning and other diagnostic procedures, detection of deep tissue tumors, even in the poorly contrasted soft tissue, may become increasingly more common. As such, a thorough understanding of the range of soft tissue neoplasms and their imaging characteristics as depicted in CBCT studies is important to ensure no potential problems are overlooked. In this case, diagnosis and treatment were able to be rendered before symptoms presented, even with the considerable size of the lesion. This case is an example of why a strong knowledge base of the normal anatomy of any and all structures within the field of view is crucial to avoid missing critical findings. Furthermore, it is important for clinicians to understand the differences in advanced imaging modalities as well as the quality of resolution and contrast each can provide for various tissue types. While a CBCT study may contain enough information to develop an initial differential diagnosis, advanced imaging may be needed for a more thorough evaluation, especially when soft tissue is involved. Interpretation of the study by board-certified oral and maxillofacial radiologists (OMFR) will help prevent missed diagnosis or misdiagnoses of incidental pathoses. Additionally, recommendations for advanced complex imaging can be provided by an OMFR.

Airway lesions require prompt diagnosis and attention in addition to subsequent coordination with multidisciplinary care providers to ensure appropriate patient management under the circumstances. Many lesions may have specific imaging characteristics that strongly indicate a diagnosis; however, advanced imaging and/or histopathologic evaluation via biopsy provides the most definitive diagnosis in addition to assisting with treatment planning. In addition, if the CBCT study does not include the entire lesion (similar to this case as noted in Fig. 2), further evaluation with a large field of view CBCT/MDCT or MRI is paramount to evaluate the extension of the lesion and the relationship to the adjacent structures.

## Data Availability

Not applicable.

## Abbreviations

CBCT: Cone beam computed tomography
MDCT: Multi-detector computed tomography
MRI: Magnetic resonance imaging
OMFR: Oral and maxillofacial radiologist
